# High-Frequency Homologous Recombination Occurred Preferentially in *Populus*

**DOI:** 10.3389/fgene.2021.703077

**Published:** 2021-08-19

**Authors:** Xining Geng, Yufei Xia, Hao Chen, Kang Du, Jun Yang, Xiangyang Kang

**Affiliations:** ^1^Henan Province Key Laboratory of Germplasm Innovation and Utilization of Eco-Economic Woody Plant, Pingdingshan University, Pingdingshan, China; ^2^Beijing Advanced Innovation Center for Tree Breeding by Molecular Design, Beijing Forestry University, Beijing, China; ^3^National Engineering Laboratory for Tree Breeding, Beijing Forestry University, Beijing, China; ^4^Beijing Laboratory of Urban and Rural Ecological Environment, Beijing Forestry University, Beijing, China

**Keywords:** homologous recombination, heteroduplex DNA, recombination events, molecular marker-assisted selection, *Populus tomentosa*

## Abstract

Homologous recombination (HR), the most significant event in meiosis, has important implications for genetic diversity and evolution in organisms. Heteroduplex DNA (hDNA), the product of HR, can be captured by artificially induced chromosome doubling during the development of the embryo sac to inhibit postmeiotic segregation, subsequently, and hDNAs are directly detected using codominant simple sequence repeat (SSR) markers. In the present study, two hybrid triploid populations derived from doubling the chromosomes of the embryo sac induced by high temperature in *Populus tomentosa* served as starting materials. Eighty-seven, 62, and 79 SSR markers on chromosomes 01, 04, and 19, respectively, that were heterozygous in the maternal parent and different from the paternal parent were screened to detect and characterize the hDNA in *P. tomentosa*. The results showed that the hDNA frequency patterns on chromosomes changed slightly when the number of SSR primers increased. The highest hDNA frequency occurred at the adjacent terminal on chromosomes, which was slightly higher than those at the terminals in the two genotypic individuals, and the hDNA frequency gradually decreased as the locus-centromere distance decreased. With the increase in the number of SSR markers employed for detection, the number of recombination events (REs) detected significantly increased. In regions with high methylation or long terminal repeat (LTR) retrotransposon enrichment, the frequency of hDNA was low, and high frequencies were observed in regions with low sequence complexity and high gene density. High-frequency recombination occurring at high gene density regions strongly affected the association between molecular markers and quantitative trait loci (QTLs), which was an important factor contributing to the difficulty encountered by MAS in achieving the expected breeding results.

## Introduction

The basic principle of molecular marker-assisted selection (MAS) is to use molecular markers closely linked to target genes to screen the selected population to realize the direct selection of genotypes during the juvenile phase and improve breeding efficiency (Collard et al., 2005; Ben-Ari and Lavi, 2012). MAS is conductive to selection during the juvenile phase (Bus et al., 2009; Karaagac et al., 2012), pyramiding disease resistance genes (Eibach et al., 2007), and as an alternative to trait selection methods that are expensive, time-consuming or technical difficulty to detect (Collard and Mackill, 2008; Varshney et al., 2014). Among them, for the selection of qualitative traits (inherited traits with Mendelian or near-Mendelian inheritance), examples of MAS have been reported for berry color, seedlessness, and flower sex in grape and disease resistance in apples and grapes (Myles, 2013), as well as major quantitative trait loci (QTL) selections, such as fruit acidity (Boudehri et al., 2009), fruit size (De Franceschi et al., 2013), rice yield (Spindel et al., 2013), and rice drought tolerance (Dixit et al., 2017). These studies have achieved significant results. For forest trees with complex genetic backgrounds, whose target traits are mostly quantitative traits, although there are hundreds of related studies, the genetic regulation of traits controlled by polygenes is more complicated, and resulting in MAS failing to achieve the expected results in tree breeding (Muranty et al., 2014).

The efficiency of MAS depends on the levels of linkage disequilibrium (LD) between molecular markers and QTLs (Muranty et al., 2014). However, in the prophase of meiosis I, the recombination between homologous chromosomes changes the linkage relationship between genes on the same chromosome and weakens the linkage between molecular markers and QTLs (Flint-Garcia et al., 2003). Therefore, the study of homologous recombination (HR) is of great significance for improving the efficiency of MAS (Boopathi, 2020). There were several methods to detect HR. In *Arabidopsis thaliana*, the use of FTL technology enables crossovers (COs) and gene conversions (GCs) to be observed under the microscope (Francis et al., 2007), and related studies based on this strategy have shown that the average frequency of GC per gene locus in a meiosis is 3.5 × 10^–4^ in the *A. thaliana qrt1* mutant (Sun et al., 2012). However, as determined with the aid of the *A. thaliana qrt1* mutant, male sterile lines and haploid induction systems, GCs occur less frequently than the abovementioned studies (Wijnker et al., 2013). The results of HR studies in *A. thaliana* indicate that HR occurs with higher frequency and a greater number of times during gamete formation (Yang et al., 2012; Sun et al., 2019). The differences in the results of these studies may be due to different research strategies and the number of markers. The occurrence of HR has a direct impact on short-term selection efficiency (McClosky and Tanksley, 2013).

Due to the characteristics of postmeiotic segregation (PMS) in plants, Dong et al. (2014) proposed a strategy to detect HR in higher plants, that is, heteroduplex DNA (hDNA), the product of HR, was detected by constructing populations from inhibited PMS using codominant simple sequence repeat (SSR) markers. This study was the first to characterize HR in *Populus* and found that the hDNA frequency of the “Zheyin3#” hybrid poplar ranges from 5.3 to 76.6%, and the hDNA frequency is closely related to the detected loci on chromosomes. However, in this study, the maximum number of SSR markers screened on the first 8 chromosomes was 21. Compared with the full length of DNA on a chromosome, the distance between adjacent SSR markers is relatively far. Obviously, the detection of relatively few SSR markers may miss the hDNA information between adjacent SSR loci. Therefore, if the number of SSR markers used for detection is increased, will it affect the hDNA frequency and recombination events (REs) detected? How does HR in plants affect the application of MAS in breeding?

In present study, applying the strategy proposed by Dong et al. (2014), the triploid populations from *Populus tomentosa* × (*P. alba* × *P. glandulosa*) obtained by doubling the embryo sac chromosomes were utilized as the research material. We screened a larger number of SSR primers distributed on chromosome 01 (Chr01) with the longest physical length, Chr04 with moderate length, and Chr19 as a sexual chromosome in *Populus*, which takes an important role on poplar breeding (Tuskan et al., 2012; Xue et al., 2020), than that in the our previous study (Geng et al., 2021) to study the effect of number of SSR markers on the hDNA frequency and REs, hDNA occurrence patterns, and discuss the influence of hDNA on MAS in *P. tomentosa*.

## Materials and Methods

### Plant Materials

Two allotriploid populations, using *P. tomentosa* with two genotypes (“3119” and “3532”) as the female parents and *P. alba* × *P. glandulosa* “YX1” as the male parent, were constructed by inducing chromosome doubling *via* high-temperature treatment at 39°C and 42°C f after female catkin pollination 16∼28 h, at which meiosis had finished, and as described in a previous study from our lab (Kang et al., 2015). We randomly selected 47 offspring from the “3119” × “YX1” hybrid and 45 offspring from the “3532” × “YX1” hybrid as materials when markers were screened on Chr01 and Chr04. When markers were screened on Chr19, 83 offspring from the “3119” × “YX1” hybrid and 70 offspring from the “3532” × “YX1” hybrid were randomly selected as materials in this study.

### Method to Detect hDNA

The hDNAs resulting from the repair of DSBs occurred at a heterozygous locus with “a” and “b” genotypes, if not properly repaired by mismatch repair machinery, will be maintained in the tetrad. Only one of the four megaspores survives, namely, the functional megaspore, and then probably forms four kinds of functional megaspores to undergo PMS. The hDNAs are separated during the first round of mitosis and form two sister chromosomes that are homoduplex and contain different genetic information at the locus. After three rounds of mitosis during polygonum-type embryo sac development, the two sister chromosomes enter two different cells and develop into a mature embryo sac with seven cells, and eight nuclei. Hybridized with a sperm cell, the embryo sac forms one normal diploid zygote. This normal process of meiosis produces haploid eggs of two genotypes, namely, “a” and “b.” It is not possible to detect hDNAs at a specific locus among diploid progeny.

Therefore, according to the strategy proposed by Dong et al. (2014), to capture hDNAs during gametophytogenesis, the first mitotic division of chromosomes in functional megaspores that carry hDNAs after the completion of meiosis must be inhibited. Through artificially inducing chromosome doubling of the embryo sac, the two sister chromosomes containing hDNA during the first round of mitosis could be reserved within the same unreduced 2n egg cell without segregation. The unreduced 2n egg cell is crossed with a normal male gamete to form an allotriploid. The heterozygous information inherited from the female parent can be detected at a codominant marker in an allotriploid, which is determined to preserve hDNA.

### DNA Extraction and Simple Sequence Repeat Markers

According to the manufacturer’s instructions, DNA was extracted from each young leaf sample using the DNeasy Plant Mini Kit (Tiangen Biotech Co., Ltd., Beijing, China). The fluorescently labeled TP-M13-SSR method (Schuelke, 2000) was employed in the present study. A forward primer at the 5′ end was attached with a universal M13 primer tail (5′-TGT AAA ACG ACG GCC AGT-3′) labeled with four fluorescent substances (6-carboxy-x-rhodamine, 6-carboxy-fluorescein, tetramethyl-6-carboxyrhodamine, or 5-hexachlorofluorescein). All primers were synthesized by Sangon Biotech (Shanghai, China) Co., Ltd. The PCR amplification protocol was as follows: 5 min at 94°C; 25 cycles of 30 s at 94°C, 30 s at the optimal annealing temperature for each SSR marker, and 30 s at 72°C; 8 cycles of 30 s at 94°C, 30 s at optimal temperature, and 30 s at 72°C; and a final extension of 8 min at 72°C. The PCR products were used for capillary electrophoresis fluorescence-based SSR analysis using the ABI 3730XL DNA Analyzer (Applied Biosystems, Foster City, CA, United States), and fragment sizes and peak areas were analyzed by GeneMarker 1.75 software (Hulce et al., 2011).

### SSR Primer Resource

Five SSR primer resources were utilized: (1) SSR primers released by the International *Populus* Genome Consortium (IPGC) for screening from the SSR database^1^; (2) SSR primers designed based on the *P. trichocarpa* genome (Yin et al., 2009) (beginning with “LG”); (3) SSR primers designed from mRNA sequences (beginning with “PTSSR” and “MB”); (4) SSR primers screened from a genetic map in *P. tomentosa* (beginning with “Ptr_”) (Du et al., 2016); and (5) SSR primers selected from a previous report (Du et al., 2012). SSR primers from the five resources mentioned above were BLAST against the genome sequence of *P. trichocarpa* v.3.0 (DOEJGI^2^) to determine the physical positions of these SSR loci. We chose all SSR primer pairs on Chr01, 04, and 19 to screen for polymorphic loci with two different alleles on homologous chromosomes of the female parent clones “3119” and “3532” and those that were different from that of the male parent “YX1.”

### Counting and Statistically Analyzing the REs

The REs were counted and statistically analyzed on these three chromosomes according to whether the hDNA occurred at an SSR locus on each chromosome. The REs were defined as follows: If hDNA occurred at two adjacent SSR loci in an inhibited PMS-type triploid, at least one recombination event was considered to occur at the two loci. In addition, due to the low hDNA frequency near the centromere, we separately counted the REs in the vicinity of the centromere; thus, when hDNA was produced at the two SSR loci on both sides of the centromere, and two REs were recorded. The ratio was calculated by dividing the number of triploid individuals with specific REs by the total number of triploid individuals in the two populations.

### Genomic Characteristic Parameters and Centromere Position Information in *Populus*

Data about chromosomal, DNA sequence and epigenetic features in *P. trichocarpa* (mainly including recombination rate, the number of MeDIP reads per kilobase of target sequence per million reads mapped, and the proportion of bases in long terminal repeat (LTR) Gypsy retrotransposons and A-, T-, or AT-rich low complexity sequences) were derived from previous research (Slavov et al., 2012). The centromeric positions on Chr01 and Chr04 are inferred from the degree of methylation in different tissues (Vining et al., 2012), and the position of the centromere is at 5.3–6.7 Mbp on Chr19 (Xin et al., 2018).

### Statistical Analysis

According to the allelic constitutions of triploid genotypes (“abc,” “abd,” “aac,” “bbc,” “aad,” and “bbd”), the hDNA frequency was calculated as follows:

(1) $$H\,F=\frac{N_{a\,b}}{N_{a\,b}+N_{a\,a}+N_{b\,b}}$$

where, HF indicates the frequency of hDNA; *N*_*ab*_ indicates the number of triploid individuals with heterozygous alleles inherited from the female parent at a specific locus; and *N*_*aa*_ and *N*_*bb*_ indicate the number of triploid individuals with homozygous alleles inherited from the female parent at the same locus.

The curves of the frequency of hDNA and linear regression plots were graphed by Origin software (version 2018), the heat map of gene density on Chr01, 04, and 19 in *P. trichocarpa* was drawn by using R language, and the analysis of *Kolmogorov-Smirnov* test (*K-S* test) linear regression and correlation was performed using SPSS version 19.0 software (SPSS Inc., Chicago, IL, United States).

To describe the concentration of REs in the population, this study used Origin 2018 (OriginLab Cor., Northampton, MA, United States) to estimate normal fitting (also known as Gaussian fitting) curve fitting parameters and test significance. The formula for normal fitting is as follows:

(2) $$y=y_{0}+\frac{A}{w\,\sqrt{\pi /2}}\,e^{-2\,\frac{{\left(x-x_{c}\right)}^{2}}{w^{2}}}$$

*x*_*c*_ indicates the mean of the normal fit; *w* represents two times the standard error σ; and FWHM is defined as the distance between the two x-coordinates when two function values are equal to half of the peak value in a curve function.

## Results

### Effect of the Number of SSR Markers on hDNA Frequency Detected in *P. tomentosa*

**FIGURE 1:**
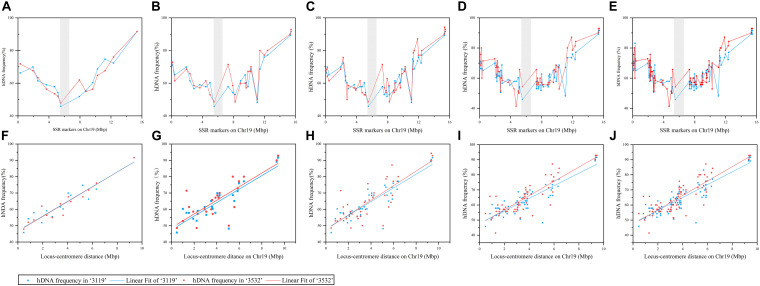
The frequency of heteroduplex DNA (hDNA) and the correlation between it and locus-centromere distance on chromosome 19 under different markers detected. The gray shadow indicated putative centromeres position on Chr19,which is between 5.3 and 6.7 Mbp (Xin et al., 2018). **(A–E)** Represented the distribution of hDNA frequency on Chr19 under 14, 24, 41, 60, and 79 markers detected, respectively; and **(F–J)** indicated the correlation analysis between hDNA frequency and locus-centromere distance on chromosome 19 under 14, 24, 41, 60, and 79 markers detected, respectively.

First, Chr19, a medium-length chromosome in *P. tomentosa*, was selected for primer screening and hDNA detection. A total of 79 pairs of SSR primers with exact chromosomal location, which were heterozygous in the maternal parent and polymorphic with the paternal parent, were screened from 639 pairs of primers. These 79 pairs of SSR primers are distributed at different positions on Chr19 in *Populus*. The centromeric position is approximately 6 Mbp on Chr19, and no suitable markers were screened on three 0.6–1.2, 5.3–6.7, and 13.6–14.4 Mbp segments because these three segments contain highly repetitive sequences or repetitions with other chromosomal sequences (Xin et al., 2018). Using formula (1) to calculate the hDNA frequency at the detection locus, the selected 79 SSR markers (Supplementary Table 1) were randomly combined into 14, 24, 41, 60, and 79 pairs of SSR primers to analyze the effect of the number of SSR molecular markers on hDNA frequency (Figure 1).

Figure 1 shows that when 14, 24, 41, 60, and 79 SSR markers were evenly distributed on the chromosome, the hDNA frequency in the distant centromeric region was significantly higher than that in the pericentromeric region, and indicating that the centromere has an important influence on the hDNA frequency. The correlation results between the hDNA frequency and the locus-centromere distance were significantly positive under the five marker combinations (*P* < 0.05), indicating that the hDNA frequency increased as the locus-centromere distance increased (Figure 1). The highest hDNA frequency (91.6% and 92.9%) was at the site between the PTSSR1123 (15.45 Mbp) and PTSSR1245 SSR markers, farthest from the centromere in *P. tomentosa* “3119” and “3532,” respectively.

### Effect of the Number of SSR Markers on REs Detected in *P. tomentosa*

**FIGURE 2:**
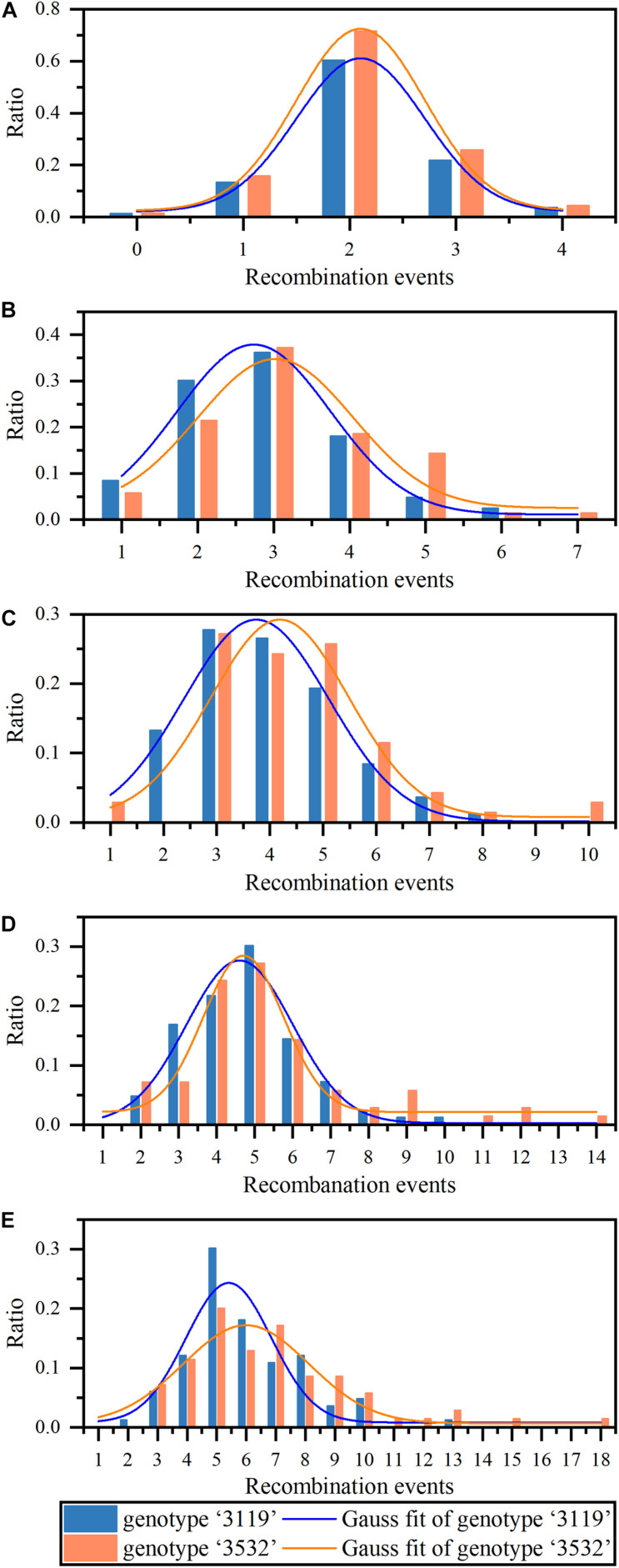
The statistics and analysis of recombination events (REs) on Chr19 under different number of markers detected. **(A–E)** Represent the proportion of recombination events in the two triploid populations under 14, 24, 41, 60, and 79 markers detected, respectively. The two curves represent gaussian fitting of the frequency distribution (normal fitting).

As determined by the *K-S* test, the REs under these five combinations of markers significantly conformed to the normal distribution in the two populations (*P* < 0.05). Based on this, formula (2) was used to perform a normal fit to the ratio of REs under five combinations of markers (Figure 2), and the normal fit results showed (Table 1) that the *x*_*c*_ and FWHM values increased with the increase in the number of markers detected, under five combinations of markers, and indicating that the average number of REs increased with the increased number of markers in a meiosis.

**TABLE 1:** Normal fitting parameters under different number of markers detected.

**Genotype**	**Number of markers**	**Mean *x*_*c*_**	***σ***	***w***	***y*_0_**	***A***	**FWHM**	***R*^2^**	**Line Integral(*x_*c*_ ± σ*)**
“3119”	14	2.1	0.605	1.209	0.021	0.894	1.424	0.996*	0.633
	24	2.7	1.011	2.021	0.011	0.931	2.380	0.992**	0.656
	41	3.7	1.363	2.727	0.001	0.994	3.211	0.957**	0.680
	60	4.6	1.398	2.796	0.003	0.959	3.292	0.957**	0.662
	79	5.4	1.443	4.247	0.008	0.850	3.399	0.842**	0.600
“3532”	14	2.1	0.605	1.209	0.025	1.060	1.424	0.996*	0.750
	24	3.0	1.023	2.046	0.025	0.827	2.409	0.846*	0.613
	41	4.2	1.295	2.590	0.008	0.924	3.049	0.809**	0.648
	60	4.7	1.059	2.118	0.022	0.699	2.494	0.933**	0.521
	79	6.0	2.123	2.887	0.007	0.879	5.000	0.857**	0.629

**Very significant difference (*P* < 0.01). *Significant different (*P* < 0.05).

Calculating the integral of the fitted curve within the range of *x*_*c*_ ± *σ*, the probability of the REs in a meiosis in *P. tomentosa* within the range of *x*_*c*_ ± *σ* could be obtained. In *P. tomentosa* “3119,” the fitting curve integral within the range of *x*_*c*_ ± *σ* was at least 0.600 (Figure 2), indicating that, under the combinations of 14, 24, 41, 60, and 79 markers, there was a > 60% possibility that 2.1 ± 0.605, 2.7 ± 1.011, 3.7 ± 1.363, 4.6 ± 1.398, and 5.4 ± 1.443 REs were detected in a meiosis, respectively. In *P. tomentosa* “3532,” the fitting curve integral within the range of *x_c_* ± *σ* was at least 0.500 (Figure 2), indicating that, under the five combinations of markers, there was a > 50% possibility that 2.1 ± 0.605, 3.0 ± 1.023, 4.2 ± 1.295, 4.7 ± 1.059, and 6.0 ± 2.123 REs were detected in a meiosis, respectively.

**FIGURE 3:**
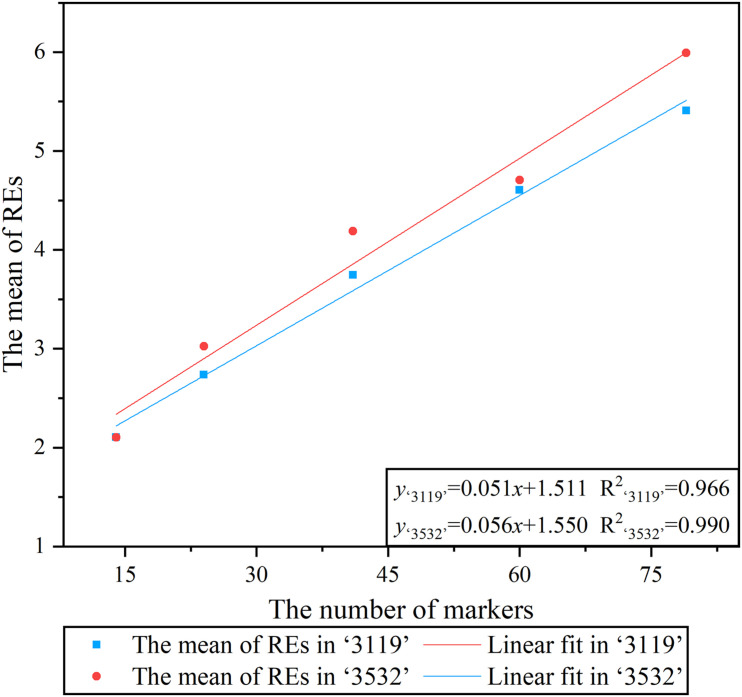
Linear regression analysis between the number of markers and the average recombination events.

The linear fitting was performed between *x*_*c*_ (average REs in a meiosis) under the five combinations of markers and the corresponding number of markers, and the result showed a significant linear relationship between them, which meant that the average REs detected in a meiosis increased as the number of markers increased (Figure 3).

### hDNA Frequency Detected on Chr01 and 04 in *P. tomentosa* Using Multiple Markers

**FIGURE 4:**
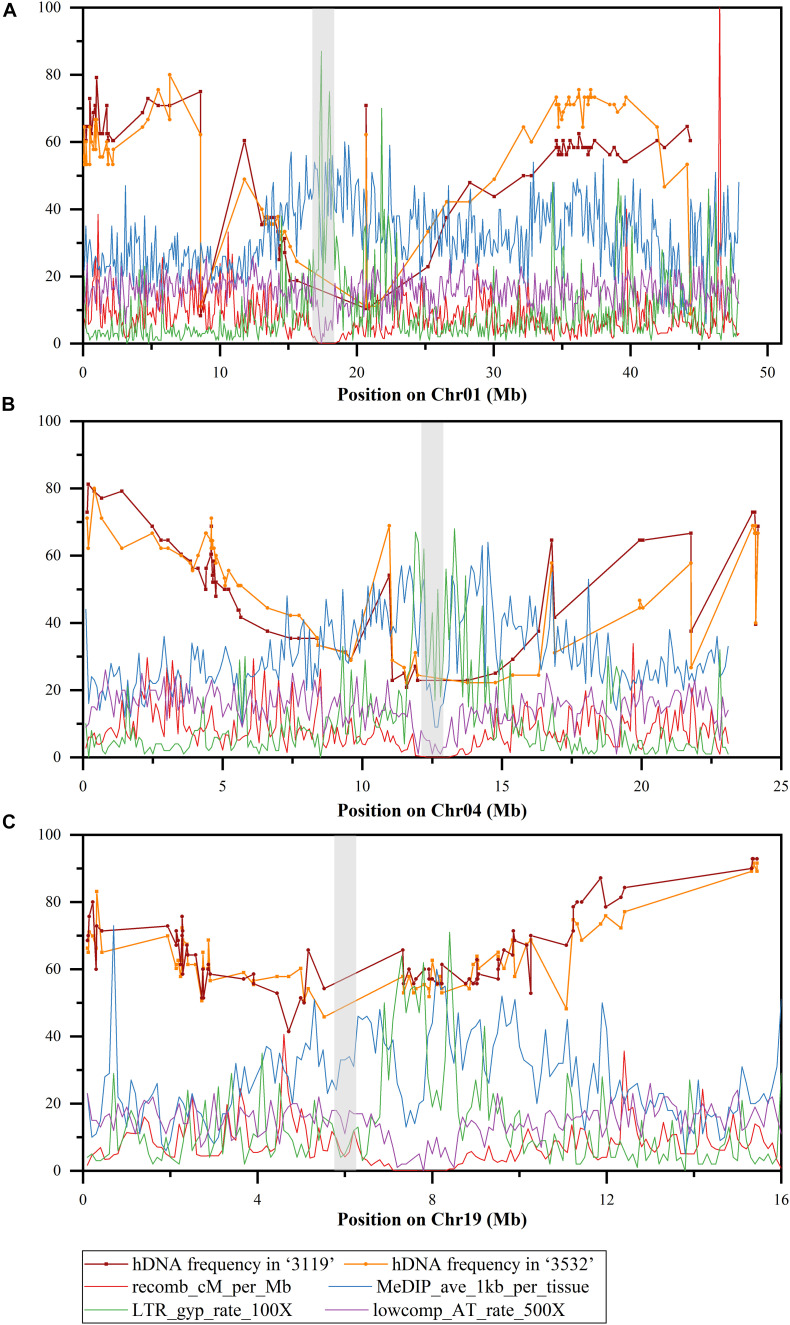
hDNA frequency (%) on Chr01, 04, and 19 under multiple markers detected (the gray boxes indicate the putative centromere region). **(A)** Chromosome 1, **(B)** chromosome 4, and **(C)** chromosome 19, recomb_cM_per_Mb indicates recombination rates estimated from linkage disequilibrium (LD); MeDIP_ave_1kb_per_tissue indicates methylation rates as measured by the number of methylated DNA immunoprecipitation (MeDIP) reads per kilobase of target sequence per million reads mapped, long terminal repeat (LTR)_gyp_rate_100× indicates the proportion of bases in LTR Gypsy retrotransposons (multiplied by 100 to equalize scales), and lowcomp_AT_rate_500× indicates the proportion of bases in A-, T-, or AT-rich low-complexity sequences (multiplied by 500 to equalize scales).

Eighty-seven SSR markers and 62 SSR markers that were heterozygous in the female parent and polymorphic with the male parent were screened on Chr01 and Chr04, respectively (Supplementary Table 1). On Chr01, the hDNA frequency in *P. tomentosa* “3119” had the highest value at the site between Ptr_4_SSR1 (0.988 Mbp) and the LG_I-1223 SSR marker, which was 79.2%; the minimum value was 8.3% at the site between PTSSR2099 (8.584 Mbp), and the GCPM_1960-1 SSR marker. The hDNA frequency in *P. tomentosa* “3532” had the maximum value at the site between GCPM_124 (6.328 Mbp) and the PTSSR2099 SSR marker, which was 80.0%; the minimum value was 8.89% at the site between Ptr_13_SSR59 (44.361 Mbp) and the Ptr_13_SSR56 SSR marker. In the pericentromere, the hDNA frequency was generally low in both individuals (Figure 4).

On Chr04, the hDNA frequency in *P. tomentosa* “3119” reached a peak at the site between GCPM_2625-1 (0.188 Mbp) and the GCPM_1116-1 SSR marker, which was 81.3%; the minimum value was 20.8% at the site between PTSSR2651 (0.208 Mbp), and the Ptr_4_SSR47 SSR marker. The hDNA frequency in *P. tomentosa* “3532” reached the peak at the site between GCPM_1116-1 (0.401 Mbp) and the ORPM_394 SSR marker, which was 80.0%; the minimum value was 22.2% at the site between PTSSR2651 (0.208 Mbp), and the Ptr_4_SSR47 SSR marker. In the pericentromere, the hDNA frequency was generally low in both individuals (Figure 4).

In addition, based on the detection of multiple markers, the hDNA frequency in the two genotypic individuals in *P. tomentosa* also showed another characteristic on the three chromosomes, namely, the highest hDNA frequency occurred at the adjacent terminal on the chromosomes, and which was slightly higher than that at the terminal (Figure 4).

**FIGURE 5:**
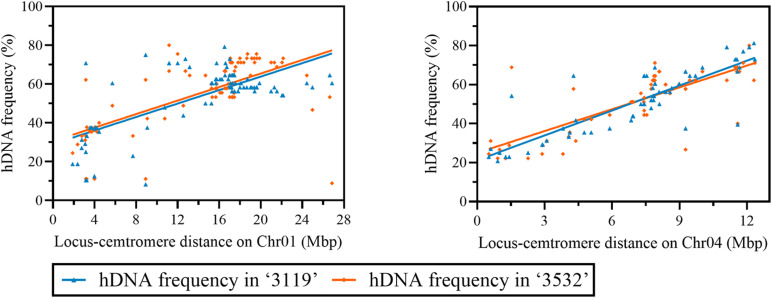
Correlation analysis between hDNA frequency and the distance from the locus to the centromere on Chr01, 04 under multiple markers.

The correlation results between the hDNA frequency and the locus-centromere distance (Figure 5) were significantly positive on Chr01 and 04 (*P* < 0.05), indicating that the hDNA frequency increased as the locus-centromere distance increased (Figure 5).

### Preference for HR in *P. tomentosa*

Compared with the recombination rate, methylation rate, LTR retrotransposon and A-, T-, or AT-rich low-complexity sequences in previous studies, the correlation between the hDNA frequencies on the three chromosomes under the multinumber markers detected and them was determined. The results (Table 2) showed that on Chr01, the hDNA frequency and methylation rate in genotype “3119” were significantly negatively correlated; the hDNA frequency in genotype “3532” had no significant correlation with the four parameters; on Chr04, the hDNA frequency in the two genotypes had a significant negative correlation with the methylation rate and LTR retrotransposon, but it was significantly positively correlated with the low-complexity sequence; and on Chr19, the hDNA frequency in the two genotypes was significantly negatively correlated with the methylation rate and the LTR retrotransposon, but it significantly positive correlated with the low-complexity sequence and recombination rate.

**TABLE 2:** Linear fitting results between hDNA frequency and genomic characteristic parameters.

**Chr**	**Genotype**	**Parameters of genomic characteristic/epigenetics**	**Slop**	**Intercept**	***R*^2^**
1	“3119”	Recombination rate	0.04	7.03	<0
		Methylation rate	–0.18	39.66	0.10**
		LTR retrotransposons	–0.03	10.15	<0
		Low-complexity sequences	0.00	15.65	<0
	“3532”	Recombination rate	0.01	8.43	<0
		Methylation rate	–0.08	34.87	0.02
		LTR retrotransposons	–0.06	11.92	0.01
		Low-complexity sequences	0.01	14.91	<0
4	“3119”	Recombination rate	0.07	5.81	0.02
		Methylation rate	–0.23	39.41	0.15**
		LTR retrotransposons	–0.37	28.68	0.21**
		Low-complexity sequences	0.12	10.11	0.16**
	“3532”	Recombination rate	0.06	6.57	0.00
		Methylation rate	–0.25	40.47	0.16**
		LTR retrotransposons	–0.32	26.26	0.15**
		Low-complexity sequences	0.12	10.07	0.15**
19	“3119”	Recombination rate	0.23	–8.05	0.13**
		Methylation rate	–0.38	53.02	0.07*
		LTR retrotransposons	–0.62	55.35	0.16**
		Low-complexity sequences	0.20	1.05	0.12**
	“3532”	Recombination rate	0.14	–2.55	0.05*
		Methylation rate	–0.28	47.13	0.04*
		LTR retrotransposons	–0.42	42.89	0.09**
		Low-complexity sequences	0.14	4.49	0.08**

**Very significant difference (*P* < 0.01). *Significant different (*P* < 0.05).

**FIGURE 6:**
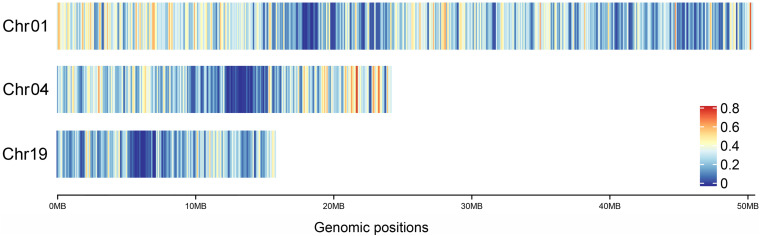
Heat map of gene density on Chr01, 04, and 19 in *P. trichocarpa*.

In addition, combined with the heat map of gene density on Chr01, 04 and 19 in *P. trichocarpa* (Figure 6), it was found that the gene density at the pericentromere was generally lower than that at the distant centromere, which was consistent with the hDNA frequency distributed on the three chromosomes. This means that the HR tended to occur in regions with high gene density.

### Detection of REs on Chr01 and 04 Using Multiple Markers in *P. tomentosa*

**FIGURE 7:**
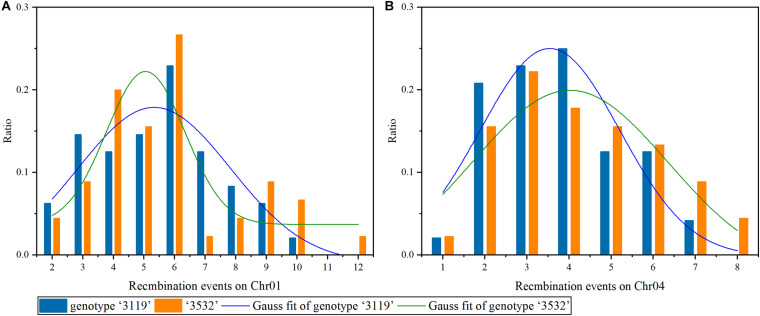
Recombination events on chromosomes 1 and 4 under multiple markers detected. **(A)** Chr01, **(B)** Chr04.

On Chr01, the maximum REs in *P. tomentosa* “3119” and “3532” were 10 and 12, respectively. The fitting curve integral within the range of *x*_*c*_ ± *σ* was at least 0.751 in *P. tomentosa* “3119” (Figure 7), indicating that there was a > 75% possibility that 5.3 ± 2.495 REs under the 87 markers were detected in a meiosis. In “3532,” the fitting curve integral within the range of *x*_*c*_ ± *σ* was at least 0.500 (Figure 7), indicating that there was a > 50% possibility that 5.1 ± 1.281 REs under the 87 markers were detected in meiosis.

On Chr04, the maximum REs in *P. tomentosa* “3119” and “3532” were 7 and 8, respectively. The fitting curve integral within the range of *x*_*c*_ ± *σ* was at least 0.706 in *P. tomentosa* “3119” (Figure 7), indicating that there was a > 70% possibility that 3.5 ± 1.659 REs under the 62 markers were detected in meiosis. In “3532,” the fitting curve integral within the range of *x*_*c*_ ± *σ* was at least 0.776 (Figure 7), indicating that there was a > 77% possibility that 4.0 ± 2.325 REs were detected in a meiosis. On both chromosomes, the maximum RE in “3532” was larger than that in “3119.”

## Discussion

### Frequency of HR During Gametogenesis in *Populus*

The genome-wide average GC frequency in *A. thaliana* is 3.5 × 10^–4^ per locus per meiosis (Sun et al., 2012). Another study estimated a frequency of 3.6 × 10^–6^ ± 2.7 × 10^–6^ for NCO–GCs (NCO: non-crossover) per site per meiosis and the frequency of 7.8 × 10^–6^ ± 5.4 × 10^–6^ for CO–GCs based on the tetrad data per site per meiosis in *A. thaliana* (Wijnker et al., 2013). In diploid transgenic *A. thaliana*, meiotic recombination frequencies at the transgenic site range from 7.4 to 20.2% (Pecinka et al., 2011). Using 8–21 SSR markers distributed on the first 8 chromosomes in *P. pseudo-simonii* × *P. nigra* “Zheyin3#” (Section *Tacamahaca*), Dong et al. (2014) reported that the frequency of hDNA, the HR product, and ranges from 5.3 to 76.6%. The similar number of SSR markers distributed on Chr01-08 and Chr19 were used to detect hDNA in the two female parents in *P. tomentosa* (Geng et al., 2021), the results showed that the frequencies of hDNA between two female parents in *P. tomentosa* ranged from 8.5 to 87.2%.

When the number of SSR markers used to detect hDNA was increased, due to the added markers covering previously undetected chromosome regions, it was found that the higher frequency of hDNA on Chr19 was 92.9% in *P. tomentosa*, and the lower frequency of hDNA was only 8.3% on Chr01 in *P. tomentosa* “3119.” Both the lowest and highest frequencies of hDNA in *P. tomentosa* (Section *Leuce*) were slightly higher than those in “Zheyin3#” poplar (Dong et al., 2014). An important reason for the difference in hDNA frequency in the same chromosomal region between poplar species and between different genotypes within the same species was related to chromosome structure distribution that inhibited HR, such as chromosomal knob, and heterochromosome and repetitive sequence (Bauer et al., 2013; Yelina et al., 2015).

### REs During Gametogenesis in *Populus*

During meiosis, the synapsis between homologous chromosomes plays an important role in the correct separation of meiosis I, and REs occur at least once during this process (Jones, 1984; Page and Hawley, 2003; Ritz et al., 2017). In *A. thaliana*, approximately 1–3 NCO–GCs and approximately 10 COs occur during a meiosis (Wijnker et al., 2013). The single-cell sequencing results in maize showed that 0–4 COs occur on a chromosome (Li et al., 2015; Luo et al., 2019). The average amount of COs on a chromosome during meiosis in rice is 1.9–3.8 (Si et al., 2015). Similarly, the results of tetrad single cell and double haploid population sequencing in barley showed that REs occur 0–4 times at Mbp resolution (Dreissig et al., 2017). However, Dong (2015) found that 1–3 REs mostly occur on each chromosome, and up to six REs are detected on Chr06 in “Zheyin3#” poplar, based on the strategy used to detect hDNA (Dong et al., 2014). Using a similar number of SSR markers to detect the REs in the two female parents in *P. tomentosa* (Geng et al., 2021), it was found that most chromosomes experienced 1–3 REs.

More REs have been reported in various studies in plants. In *A. thaliana*, meiosis produces approximately 657 REs, of which > 90% of the REs are GCs and 12.6, 13.7, 13.7, 8.3, and 10 COs occur on Chr01-05, respectively, while 152.7, 88.8, 128.7, 94.4, and 148.8 gene conversion events occur on Chr01-05, respectively (Yang et al., 2012). Using next-generation sequencing in *Populus*, the results showed that an average of 684.1 GCs occurred in a meiosis in *P. simonii*, and an average of 924.7 GCs occurred in a meiosis in *P. deltoides*. The average COs in a meiosis occur 27.3 and 34.8 times in the parents, respectively (Tao et al., 2018), which is roughly similar to the COs in *A. thaliana* (Yang et al., 2012). The results of REs detected in *P. tomentosa* using more SSR markers in the present study showed that REs significantly increased on a chromosome as SSR loci detected increased during female gametogenesis. In the present study, SSR markers screened to detect alleles in parents should satisfy the following conditions: (1) two different alleles detected in homologous chromosomes of the female parent; (2) different alleles from female parent of the male parent; and (3) the locations of SSR markers are clear. Limited by the conditions, it’s difficult to screen more SSR markers. Using 87, 62, and 79 SSR markers in the study, up to 12, 8, and 18 REs could be detected on chromosomes Chr01, 04, and 19, respectively. Since the 62–87 SSR markers selected only covered a small part of the chromosome in *P. tomentosa*, it was speculated that there should be more REs on chromosomes to produce hDNA during gametogenesis. Therefore, the acquisition of high-throughput markers is the key to comprehensively evaluate REs in the overall genome in *Populus*. Recently, using genome sequencing and high-precision polyploid genome assembly (Zhou et al., 2020; Yu et al., 2021) provides technical support for the acquisition of high-throughput SNPs, which is expected to solve the problem.

### Preference of HR in *P. tomentosa*

In higher plants, meiotic recombination occurs unevenly on chromosomes (Lambing et al., 2017). In *A. thaliana*, rare REs are detected at pericentromere (Wijnker et al., 2013; Sun et al., 2019). Tetrad analysis by single-microspore sequencing in maize reveal that COs are unevenly distributed throughout the genome, recombination is unlikely to occur near centromeres (Li et al., 2015). There are numerous recombination hot spots and cold spots detected in rice, and the cold spots are located on centromeres or pericentromeric regions (Si et al., 2015). The result that the positive correlation between the hDNA frequency on the chromosome and locus-centromere distance are detected in *Populus*, using a dozen or so of SSR markers (Dong et al., 2014; Geng et al., 2021). In the present study, the hDNA frequency detected in *P. tomentosa* also showed the same pattern when the number of SSR markers was increased. The same results revealed among plant species are attributed to heterochromosomes, gene poor, and the enrichment of LTR retrotransposons in the pericentromere inhibiting meiotic recombination (Henderson, 2012).

It is worth noting that the hDNA frequency at the chromosome terminal was lower than that at the adjacent terminal on chromosomes, using 87, 62, and 79 SSR markers to analyze the hDNA patterns on Chr01, 04, and 19, respectively, in *P. tomentosa*. While relevant phenomena were hardly observed when a dozen or so of SSR markers were screened in *Populus* (Dong et al., 2014; Geng et al., 2021). Linked-read sequencing of gametes in maize show the similar results (Sun et al., 2019). The phenome that the recombination frequency at the adjacent terminal is higher than that at the chromosome terminal is due to the telomere that protects chromosomes containing repetitive sequences and a high level of heterochromosomes, resulting in reduced recombination frequency (Saintenac et al., 2009; Henderson, 2012), and to the subtelomeric regions that are enriched with genes and DNA transposons, with a low degree of methylation, where meiotic recombination occurs frequently in plants (Fayos et al., 2019).

Studies reported that the epigenetic characteristic factors that suppress HR frequency include the degree of methylation, LTR retrotransposons, heterochromatin regions and chromosomal knobs (Bauer et al., 2013; Yelina et al., 2015), and which is similar with the present study. The preference for hDNA in *P. tomentosa* (Section *Leuce*) was generally similar with that in “Zheyin3#” poplar (Section *Tacamahaca*) (Dong et al., 2014), which is related to conservative within genus *Populus*. In *A. thaliana*, REs preferentially target at the gene body (Wijnker et al., 2013). In maize, high-frequency recombination occurs at the 5′ and 3′ ends of the gene (Li et al., 2015). In present study, it was found that there was a certain relationship between hDNA frequency and gene density, and that the hDNA frequency was relatively higher in gene-intensive regions in *P. tomentosa*. Recombination prefers to target gene-rich tracts, thereby increasing copy number variations (Lu et al., 2012; Wijnker et al., 2013) and causing more phenotypic variations, and which accelerates the evolution of plants to adapt to changing environments.

In addition, studies have shown that recombination hotspots also prefer simple sequences, such as AT-rich, CTT-repeat, and CNN-repeat motifs (Lawrence et al., 2017). However, recombination preferences for simple sequences are different among species. In *A. thaliana* and *Solanum lycopersicum*, recombination prefers poly-A repeat motifs (Choi et al., 2013; Wijnker et al., 2013; Demirci et al., 2017). Meiotic recombination in maize prefers GC sequences (Rodgers-Melnick et al., 2015; Sun et al., 2019). High-frequency recombination in rice occurs at the low CA dinucleotide region (Demirci et al., 2018). In this study, the hDNA in *P. tomentosa* tended to target A-, T- or AT-rich low-complexity sequences. Different results among plant species are related to variations of plant species.

### Effects of HR on MAS

In contrast to crops that have undergone long-term domestication, forest trees have more natural genetic diversity, and leading to low-level LD (Neale and Savolainen, 2004). The LD value within the forest tree population decays to insignificance within 1∼2 kbp (Neale and Ingvarsson, 2008; Neale and Kremer, 2011). In the natural population in *P. trichocarpa*, the average decay range of LD in the genome is 3–6 kbp, and the average levels of LD in or near the gene are lower than those of the whole genome, while the levels of LD are high in the centromere and on chromosome segments with low gene density (Slavov et al., 2012). In the natural population in *P. tomentosa*, the LD declines within 1,200 bp (Du, 2014). Obviously, unless the molecular marker is within the gene or extraordinarily close to the target gene, it is difficult to ensure that the physical distance between the marker and the target gene is less than 1 kbp. Based on the HR results in *P. tomentosa*, there should be more REs on the chromosome to yield hDNA during gametogenesis, HR tended to occur the segments that were far from centromeres on chromosomes and that were enriched with genes, and HR maintained high frequency in the population. High-frequency HR that occurred in gene-intensive areas seriously affected the association between molecular markers and QTLs and aggravated the decay of LD, which is an important reason why it was difficult for MAS to achieve the expected breeding results.

## Data Availability Statement

The original contributions presented in the study are included in the article/Supplementary Material, further inquiries can be directed to the corresponding author.

## Author Contributions

XG and XK designed the experiments and wrote the manuscript. XG, YX, HC, and KD performed the experiments and analyzed the data. JY revised the manuscript. All authors contributed to the article and approved the submitted version.

## Conflict of Interest

The authors declare that the research was conducted in the absence of any commercial or financial relationships that could be construed as a potential conflict of interest.

## Publisher’s Note

All claims expressed in this article are solely those of the authors and do not necessarily represent those of their affiliated organizations, or those of the publisher, the editors and the reviewers. Any product that may be evaluated in this article, or claim that may be made by its manufacturer, is not guaranteed or endorsed by the publisher.

## Footnotes

1
http://web.ornl.gov/sci/ipgc/ssr_resources.htm

2
http://www.phytozome.net/poplar

## Supplementary Material

Supplementary Table 1 Primer information to detect hDNA in *P. tomentosa*.
